# Extreme Metabolic Alkalosis Caused by Temporary Jejunostomy—A Case Report and Physiopathological Insights

**DOI:** 10.3390/diagnostics16030443

**Published:** 2026-02-01

**Authors:** Narcis-Valentin Tănase, Ștefan-Antoniu Aionese, Andrei Tănase, Luana-Maria Gherasie

**Affiliations:** 1 Department of Anaesthesia and Intensive Care Medicine, Carol Davila University of Medicine and Pharmacy, 050474 Bucharest, Romania; 2 Clinic of Anaesthesia and Intensive Care Medicine, Dr. Carol Davila Central University and Emergency Military Hospital, 010825 Bucharest, Romania; 3 Faculty of Medicine, Carol Davila University of Medicine and Pharmacy, 050474 Bucharest, Romania; tanase_andrei16@yahoo.com; 4 Department of Otorhinolaryngology, Carol Davila University of Medicine and Pharmacy, 05047 Bucharest, Romania; luana-maria.bujor@drd.umfcd.ro; 5 Department of Otorhinolaryngology, “Prof. Dr. Dorin Hociota” Institute of Phonoaudiology and E.N.T. Functional Surgery, 061344 Bucharest, Romania

**Keywords:** metabolic alkalosis, severe, jejunostomy, adult

## Abstract

**Background and Clinical Significance**: Metabolic alkalosis is the most common acid–base disturbance in hospitalized and critically ill patients, with extreme alkalemia (pH > 7.65) linked to mortality rates exceeding 80%. Jejunostomy-related intestinal losses can lead to severe hypochloremic metabolic alkalosis, a rare but life-threatening condition. This case report highlights the clinical presentation, diagnostic approach, physiopathology, management, and outcome of a patient with extreme metabolic alkalosis induced by a temporary jejunostomy. **Case Presentation**: We report the case of a 72-year-old female who presented with severe alkalemia, seizures, and signs of profound dehydration following extensive enteral resection with end-jejunostomy. Serial arterial blood gas and serum electrolyte monitoring guided treatment, prompting the initiation of an aggressive chloride-based rehydration protocol. Concurrent evaluations revealed renal impairment and an intercurrent infection. Initial tests revealed extreme metabolic alkalosis (pH 7.757, HCO_3_^−^ 72.7 mmol/L) with severe hypochloremia, hypokalemia, and acute kidney injury. Administration of approximately 5 L of isotonic saline with added potassium chloride over the first 6 h led to rapid improvement in pH to near-normal levels. Over the following six days, continued electrolyte correction restored physiological acid–base balance and renal function. After achieving metabolic stabilization, the jejunostomy was surgically reversed. **Conclusions**: Extreme metabolic alkalosis secondary to jejunostomy is rare but potentially fatal. Prompt recognition of chloride-responsive alkalosis and rapid initiation of aggressive volume and electrolyte replacement are essential for survival. Definitive management requires addressing the underlying cause, such as restoration of gastrointestinal continuity, to prevent recurrence.

## 1. Introduction

In the specialized literature, there is notable variability in the findings of epidemiological studies on acid-base disorders. Metabolic alkalosis is the most prevalent acid-base imbalance in hospitalized patients (accounting for nearly 51% of all acid-base disorders), while in intensive care, the prevalence is about 85% [1,2]. An increase in pH above 7.55 defines extreme metabolic alkalosis and exhibits a mortality rate of over 80% [3,4].

Loss of gastric acid induced by vomiting or nasogastric tube drainage and the use of diuretics, especially loop or thiazide diuretics, causes volume contraction and is the most common cause of metabolic alkalosis [5]. The causes of metabolic alkalosis include ingestion or infusion of bicarbonate (milk-alkali syndrome) or relative acid loss, which, according to intravascular volume, is classified as chloride-responsive or chloride-resistant alkalosis.

Loss of enteral fluid through a jejunostomy or ileostomy is one of the causes of hypochloremic metabolic alkalosis, which typically occurs following significant chloride losses, an essential electrolyte for maintaining acid-base balance. Treatment consists of administering chloride, correcting electrolyte imbalances, and, if possible, restoring transit [6].

Several cases of severe metabolic alkalosis have been reported in the literature to date. Notable cases with a pH value greater than 7.75 include one with a pH of 7.93 due to uncontrollable vomiting [7], one with a maximum pH of 7.91 due to severe pyloric obstruction [8], and one with a pH of 7.87 caused by combined losses through jejunostomy and nasogastric tube drainage [9].

In this report, we present the case of a 72-year-old female patient with extreme metabolic alkalosis (pH 7.757) caused by the intestinal loss of acids and electrolytes through a temporary jejunostomy performed as a result of extensive enteral resection, which will be described further below.

## 2. Case Presentation

We present the case of a 72-year-old female patient who was admitted to the emergency department in a severely deteriorated overall condition, with generalized tonic-clonic seizures. Clinical examination revealed a conscious, agitated patient in critical condition, with dry, pale, and dehydrated skin; a heart rate of 82 beats per minute, a blood pressure of 119/62 mmHg, a sinus rhythm, and a temperature of 36.3 °C.

The patient’s recent history indicated that ten days prior, she had presented with nausea accompanied by vomiting, diffuse abdominal pain, and absence of bowel movement for two days. She underwent an emergency exploratory laparotomy ten days before, which revealed adhesion syndrome, multiple fecalomas throughout the entire colonic framework, and dilated ileo-jejunal loops with irreversible ischemia in an extensive non-viable portion, lacking peristaltic movement, and volvulated up to the last ileal loops. Successful adhesiolysis and wide enterectomy were performed, along with a terminal left iliac jejunostomy. The postoperative course was favorable, with the patient in good general condition, afebrile, stable vital parameters, and the left iliac jejunostomy patent, draining liquid content. The patient additionally had a notable medical history, which included a surgically treated cervical neoplasm via hysterectomy with bilateral annexectomy in 2011, followed by radiotherapy, bilateral inguinal hernias, a thyroid nodule, and erythematous gastritis. The surgical and radiotherapeutic management of the cervical neoplasm could have contributed to the development of the adhesion syndrome identified during her episode of intestinal obstruction.

In the current episode, the arterial blood gas (ABG) analysis in the emergency department revealed severe alkalemia (pH 7.663), with a PaCO_2_ of 61.7 mmHg and an HCO_3_^−^ level of 76.0 mmol/L, consistent with profound metabolic alkalosis. Serum electrolyte levels showed the following values: severe hyponatremia (Na^+^ 121 mmol/L), moderate hypokalemia (K^+^ 2.2 mmol/L), and ionic hypocalcemia (Ca^2+^ 3.1 mmol/L). Given the gravity of her clinical presentation and the severe nature of her symptoms, the decision was made to transfer the patient to the intensive care unit for closer monitoring and intensive management.

Upon admission to the intensive care unit, analyses confirmed severe hypochloremic metabolic alkalosis (pH 7.757, PaCO_2_ 46.8 mmHg, HCO_3_^−^ 72.7 mmol/L, and base excess (BE) of 46.9 mmol/L), with worsening alkalemia and clinical signs of severe hypovolemia (pale, dry, and dehydrated skin) and acute kidney failure (Acute Kidney Injury Network AKIN stage 3).

An aggressive treatment plan was initiated to correct the metabolic alkalosis and electrolyte imbalances, involving the administration of 5000 mL of 0.9% NaCl (sodium chloride) plus 20 mEq of KCl (potassium chloride) per 500 mL in the first six hours. Additionally, MgSO_4_ (magnesium sulfate) and calcium were administered. Within the first six hours, alkalemia improved, with the pH approaching the upper limit of the physiological range (7.46); however, the metabolic alkalosis persisted with measured values of PaCO_2_ at 64.3 mmHg, HCO_3_^−^ at 44.0 mmol/L, and BE at 22.4 mmol/L. Fluid administration continued with an additional 500 mL of 0.9% NaCl and 1000 mL of 10% glucose over the first 24 h (as maintenance calories while *nil per os* status).

During the second day, the correction treatment was sustained with the administration of 1500 mL of standard saline solution, 1000 mL of 10% glucose, and 500 mL of Sterofundin (a balanced saline solution with an electrolyte composition similar to that of human plasma). Laboratory analyses confirmed renal dysfunction, revealing serum urea at 127 mg/dL (reference range 17 mg/dL), creatinine at 5.37 mg/dL (reference range 0.51–0.95 mg/dL), and serum albumin at 2.1 g/L. Additionally, a urinary tract infection caused by E. coli was detected, prompting initiation of ciprofloxacin treatment.

By the end of the second day in ICU, the ABG results indicated a return toward a more normalized alkaline state, with a pH of 7.57, PaCO_2_ of 45.8 mmHg, HCO_3_^−^ at 42.7 mmol/L, and BE at 20.5 mmol/L. However, electrolyte imbalances remained evident, with potassium at 2.9 mmol/L, sodium at 133 mmol/L, and chloride at 81 mmol/L.

In the following days, ongoing treatment focuses on restoring the hydro-electrolytic balance. Starting on the third day, a consistent improvement in acid-base balance parameters and electrolyte levels was noted, as illustrated in Table 1 and the accompanying graphs.

On the sixth ICU day, ABG revealed a return to normal physiological parameters: pH 7.46, PaCO_2_ of 40 mmHg, HCO_3_^−^ of 27.8 mmol/L, BE of 4.7 mmol/L, K^+^ at 4.0 mmol/L, Na^+^ at 134 mmol/L, ionic Ca^2+^ at 4.0 mmol/L, and Cl^−^ at 99 mmol/L. The patient was transferred from the intensive care unit to the surgical department, where surgical intervention was to be performed two days later (and nine days after the current admission) to restore gastrointestinal continuity through a manual lateral-lateral entero-enteric anastomosis. The purpose of this intervention was justified by the need to reintroduce the ileostomy into the transit system, thereby breaking the vicious cycle of acid-base disturbances induced by massive losses of electrolytes and acids.

Postoperatively, the patient’s evolution was favorable: afebrile and hemodynamically stable, with the resumption of intestinal transit and healing of the surgical wound.

**Figure 1:**
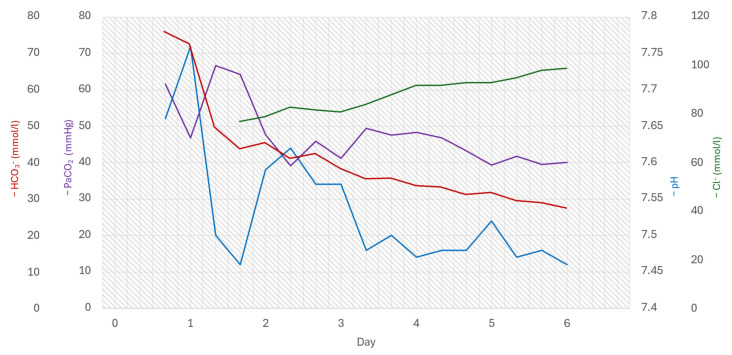
Time evolution of pH, PaCO_2_, HCO_3_^−^ and Cl^−^.

As adjunctive treatment for intestinal losses of H^+^ and Cl^−^, the patient was prescribed a proton pump inhibitor (pantoprazole) from the first ICU day, and, starting on the second day, an opioid agonist (loperamide) was administered throughout the hospitalization period. Figure 1 illustrates the dynamic progression of pH, PaCO_2_, HCO_3_^−^, and Cl^−^ levels during hospitalization.

## 3. Discussion

### 3.1. Pathophysiology of Jejunostomy-Induced Metabolic Alkalosis

The loss of hydrochloric acid from the stomach through externalization leads to a decrease in the concentration of H^+^ ions in the blood. Simultaneously, an electrolyte imbalance occurs due to the loss of essential electrolytes, such as chloride. In the presented case, the intestinal losses that cause metabolic alkalosis are due to jejunostomy.

Under normal conditions of gastrointestinal integrity, approximately 8–10 L of fluids are secreted daily: the stomach secretes gastric acid (rich in H^+^ and Cl^−^); the pancreas secretes bicarbonate (rich in HCO_3_^−^); and the intestines secrete fluids rich in Na^+^, Cl^−^, HCO_3_^−^, and water. Most of these secretions are reabsorbed by the distal small intestine and colon (water, Na^+^, and Cl^−^) [10].

The dynamic acid-base balance is maintained by the equal loss/secretion of H^+^ from the stomach and HCO_3_^−^ from the small intestine. In the case of a jejunostomy due to various causes (ischemia, necrosis, obstruction, mesenteric infarction), HCO_3_^−^ is no longer secreted by the small intestine in the same proportions as H+ secreted by the stomach. Additionally, Cl^−^ (the other component of gastric juice) is not reabsorbed as effectively by the small intestine, leading to a relative loss of Cl^−^ compared to other electrolytes and creating conditions that favor the development of metabolic alkalosis.

This clinical case represents one of the most severe examples of metabolic alkalosis documented in the literature, characterized by the unique mechanism underlying the onset of acid-base imbalances and the gradual yet favorable progression in response to appropriately instituted aggressive treatment. Additionally, the early and somewhat forced reintegration of the jejunostomy significantly curbed the pathophysiological process and, consequently, led to remission of the disturbances.

### 3.2. Comparison with Previously Reported Cases

Severe metabolic alkalosis caused by jejunostomy is rare, with only a few adult cases reported. Table 2 summarizes the most relevant cases in the literature [9,11,12], including presentations with extreme alkalemia (pH > 7.65), similar to the present patient. These cases highlight shared features such as profound hypovolemia, severe hypochloremia, hypokalemia, and an elevated bicarbonate level. Survival in cases with a pH > 7.70 is uncommon, emphasizing the severity of the condition and the importance of timely recognition and management.

Notably, several of the reported cases involve extreme alkalemia (pH > 7.65), which carries a very high mortality risk. Standard features include profound volume depletion, hypokalemia, hypochloremia, and often secondary acute kidney injury, given the chloride depletion alkalosis physiology [13]. Treatment in all cases centered on aggressive volume and chloride repletion (usually IV saline with potassium supplementation), removal or reduction in the offending fluid loss (e.g., clamping tubes or surgical reversal), and adjunctive measures such as acetazolamide or proton-pump inhibitors, as needed. Outcomes were favorable in these reported cases with prompt recognition and management.

All patients presented with hypovolemic, chloride-responsive metabolic alkalosis secondary to GI losses. Each case required intensive care support due to the severe alkalemia (some with pH > 7.70). Notably, the highest reported survivable pH was 7.87 in cases with combined jejunostomy and NG tube losses. Consistent clinical findings included neuromuscular irritability (e.g., seizures, tetany), arrhythmia risk (prolonged QT or dysrhythmia), and electrolyte derangements (often K^+^ < 3 mEq/L, Cl^−^ < 85 mEq/L). Management uniformly involved replacing volume and lost electrolytes (particularly chloride and potassium) and addressing the source of loss. In two cases, pharmacologic suppression of gastric secretions (proton-pump inhibitors or H_2_-blockers) and/or acetazolamide (to enhance renal bicarbonate excretion) were used adjunctively. Definitive treatment may require surgical or procedural intervention—for example, reinfusing or stopping drainage, or re-establishing intestinal continuity (as performed in the present case to cure the alkalosis).

### 3.3. Mixed Acid-Base Disturbances in the Current Case

In our case, the elevated pH level (7.757) indicates severe alkalemia. The increased bicarbonate level of 72.7 mmol/L suggests a metabolic disorder as the primary cause, while the elevated PaCO_2_ of 46.8 mmHg indicates hypoventilation as the compensatory mechanism.

The subsequent step involved evaluating and quantifying the compensatory response. In instances of primary metabolic alkalosis, the compensatory response is manifested as hypoventilation, leading to an increase in PaCO_2_. By calculating the expected PaCO_2_ using the formula PaCO_2_ = (0.7 × [HCO_3_^−^]) + 20 ± 5 mmHg [14], we obtained a value of 71 ± 5 mmHg. Upon comparison, we observed that the measured PaCO_2_ (46.8 mmHg) was lower than the expected value, indicating that respiratory compensation was limited and ineffective. Furthermore, we noted that the expected PaCO_2_ after 6 h of intensive care admission is 50.8 mmHg, while the measured PaCO_2_ is 64.3 mmHg, indicating residual compensatory hypoventilation [14].

The calculated anion gap (AG) was 9.0 mmol/L; however, the patient is hypoproteinemic, with an albumin level of 2.1 g/dL. The corrected anion gap value, adjusted for albumin, yielded an AG_cor of 14 mmol/L, which was at the upper limit of normal (10–14 mmol/L) [14], suggesting the possible presence of an added metabolic acidosis.

A potential explanation for concomitant metabolic acidosis was renal dysfunction. Due to severe hypovolemia secondary to massive fluid loss through the jejunostomy, the patient develops acute prerenal kidney failure (AKI).

The fundamental pathophysiological mechanism in this reported case is characterized by profound chloride depletion. This depletion disrupts the strong ion difference and subsequently leads to metabolic alkalosis [4,13,14].

A limitation of this case report is the relative scarcity of significant laboratory values. Likely due to transient logistical issues within the central laboratory of the hospital, we obtained only one urinary chloride value from D2, which was notably low at 8.0 mmol/L. The creatinine value on D0 was 4.91 mg/dL (reference range 0.51–0.95), and the serum magnesium values were recorded at 2.7, 3.05, and 2.09 mg/dL (reference range 1.9–2.5 mg/dL) on D1, D3, and D7, respectively. Unfortunately, serum phosphorus was measured only once (3.1 mg/dL on day 8; reference range 2.5–4.5 mg/dL).

### 3.4. Clinical Implications and Management Considerations

Fluid losses through the jejunostomy caused, in this case, profound metabolic alkalosis. The rapid identification of the etiology and the initiation of aggressive hydro-electrolytic resuscitation treatment within the first six hours led to the stabilization of the patient’s overall condition. Moreover, conservative correction over the subsequent 6 days enabled the patient’s transfer to the surgical ward, where surgical reintegration of the jejunostomy was performed to interrupt the original mechanism of disturbance. Without surgical intervention, the intestinal losses of acids and electrolytes would have continued, leading to future decompensations.

## 4. Conclusions

Alkalemia with a pH above 7.65 is considered extreme and has a mortality rate exceeding 80%. Immediate identification and treatment are essential for the patient’s survival. Based on clinical examination and ABG analyses, metabolic alkalosis and responsiveness to chloride can be rapidly identified.

Our 72-year-old patient, with a history of neoplasia, presented with severe alkalemia and a pH of 7.76. Her recent history of jejunostomy and clinical signs of dehydration suggested chloride sensitivity and the need for intensive care unit admission. Aggressive rehydration with chloride and electrolytes stabilized the patient’s overall condition; after six hours, her pH was brought to the upper limit of the physiological range. Over the following 6 days, continued treatment led to the resolution of alkalosis, and the patient was transferred to the general surgery ward.

Intestinal losses through jejunostomy are a less common cause of metabolic alkalosis, yet they can lead to a severe form of the condition due to their nature. Following stabilization and resolution of the acute episode, addressing the underlying cause by restoring intestinal transit will help prevent potential future recurrences. The patient underwent surgery; the jejunostomy was reversed, and gastrointestinal transit was restored. With favorable progression, remaining afebrile, and gradually resuming intestinal transit and tolerance to digestion, the patient was discharged uneventfully.

## Figures and Tables

**Table 1:** The values of pH, PaCO_2_, HCO_3_^−^, Na^+^, Cl^−^, K^+^ and ionized Ca^2+^ during the hospital admission. The first three serum chloride determinations are not reported due to values below the reportable range (NA—not available).

Day	pH	PaCO_2_	HCO_3_^−^	Na^+^	Cl^−^	K^+^	Ionized Ca^2+^
		mmHg	mmol/L	mmol/L	mmol/L	mmol/L	mg/dL
	(7.35–7.45)	(35–45)	(22–26)	(135–145)	(98–107)	(3.5–4.5)	(4.6–5.4)
0	7.66	61.7	76	121	NA	2.2	3.1
1	7.76	46.8	72.7	120	NA	3.5	2.9
	7.5	66.7	49.9	128	NA	3.0	3.9
	7.46	64.3	44	130	77	4.1	3.9
2	7.59	47.7	45.6	132	79	3.0	4.2
	7.58	49.3	46.7	130	78	4.6	4.0
	7.62	39.2	41.4	132	83	2.9	4.3
	7.55	47.6	42.3	131	83	2.8	4.4
	7.57	45.8	42.7	132	82	2.9	4.3
3	7.57	41.2	38.6	133	81	3.3	NA
	7.58	42.1	40.6	131	86	3.5	4.2
	7.48	49.4	35.7	126	84	3.3	4.1
	7.5	47.6	35.9	130	88	3.4	4.2
4	7.47	48.3	33.8	132	92	3.3	4.1
	7.48	46.9	33.5	132	92	3.2	4.1
	7.48	43.3	31.5	131	93	3.8	4.1
5	7.52	39.3	32.1	130	93	3.4	4.1
	7.47	41.7	29.8	131	95	3.5	4.1
	7.48	39.6	29.2	132	98	4.5	4.2
6	7.46	40	27.8	134	99	4.0	4.0

**Table 2:** Published cases of metabolic alkalosis due to jejunostomy-related fluid loss in adults. Each case details the patient’s demographics, cause of the jejunostomy, severity of alkalosis, clinical presentation, key laboratory findings, treatment, and outcome.

Reference (Year)	Patient	Jejunostomy Context	Highest pH	Presentation	Notable Labs	Treatment	Outcome
Tugrul et al., 2010 [9]	Middle-aged Male	Jejunostomy after colectomy (colon cancer); NG tube in place (post-op)	7.87	Altered mental status; suspected from “life-incompatible” alkalemia	K^+^ 2.4 mEq/L; Cl^−^ 72 mEq/L (hypochloremic alkalosis) HCO_3_^−^ 48 mEq/L	Aggressive IV fluids and electrolyte repletion (chloride and K^+^); supportive care (details not fully described)	Survived—alkalosis corrected with therapy (first reported survival at such high pH)
Sanon et al., 2019 [11]	82-year-old Female	Open feeding jejunostomy + venting gastrostomy (for gastric ulcer perforation)	7.70	Syncope; confusion; arrhythmia (atrial-paced rhythm noted)	K^+^ 2.5 mEq/L, Cl^−^ 60 mEq/L, HCO_3_^−^ > 50 mEq/L; glucose 466 mg/dL	IV normal saline + KCl repletion; acetazolamide; proton pump inhibitor (Pantoprazole)	Marked improvement in 48 h (pH down to ~7.57; HCO_3_^−^ ~44); patient stabilized for further care
Turner et al., 2024 [12]	55-year-old Male	Gastrojejunostomy feeding tube-dependent patient; inadvertent continuous gastric drainage into Foley bag for weeks	7.61 (venous blood gas)	Lethargy, altered mental status; bradycardia (HR ~56) with prolonged QT ~690 ms (risk of arrhythmia); one brief seizure in ICU	Na^+^ 120 mEq/L, Cl^−^ 50 mEq/L, K^+^ 2.0 mEq/L, HCO_3_^−^ ~77 mEq/L (marked alkalosis); acute kidney injury (Cr ~0.54 mg/dL from baseline 0.25)	Stopped gastric losses (disconnected/clamped G-tube); IV saline and electrolyte infusions (K^+^, Mg^2+^, Ca^2+^) in ICU; monitored on telemetry; gastrojejunostomy tube repositioned by IR on day 2	Gradual recovery; electrolytes and ECG normalized within 24–48 h; alkalosis resolved by day 7 with supportive care; discharged home in stable condition
current case	72-year-old Female	Temporary end-jejunostomy after emergency enterectomy (ischemic non-viable mid-ileum/jejunum resected for acute obstruction)	7.76	Agitation, generalized seizures on presentation; signs of severe dehydration (dry mucosa, poor turgor) and hypotonicity	ABG: pH 7.757, pCO_2_ 46.8 mmHg, HCO_3_^−^ 72.7 mM; Na^+^ 121 mM, K^+^ 2.2 mM, Cl^−^ (unmeasurably low; hypochloremia), ionized Ca^2+^ 3.1 mg/dL; BUN 127 mg/dL, Cr 5.37 mg/dL (AKI)	Aggressive chloride repletion: ~5 L 0.9% saline + KCl in first 6 h; continued IV saline + balanced fluids, K/Mg/Ca over 6 days in ICU; IV antibiotics for intercurrent UTI. No HCl needed (responsive to saline)	pH corrected to <7.5 within 6 h; metabolic alkalosis fully resolved by day 6. Jejunostomy was surgically reversed after stabilization, restoring GI continuity and preventing recurrence

NG = nasogastric. IR = interventional radiology. UTI = urinary tract infection.

## Data Availability

The data supporting the findings of this study are derived from the patient’s medical record. Due to privacy and ethical restrictions, these data are not publicly available.

## Abbreviations

The following abbreviations are used in this manuscript:

ABG	Arterial blood gas
AKI	Acute Kidney Injury
AKIN	Acute Kidney Injury Network
A_Tot_	Total Concentration of Weak Acids
BE	Base excess
Ca^2+^	Ionized Calcium
Cl^−^	Chloride
ECG	Electrocardiogram
HCO_3_^−^	Bicarbonate
HR	Heart rate
ICU	Intensive care unit
IR	Interventional radiology
K^+^	Potassium
MgSO_4_	Magnesium sulfate
Na^+^	Sodium
NG	Nasogastric
PaCO_2_	Partial Pressure of Carbon Dioxide
pH	Potential of Hydrogen
PP	Proton pump (as in proton pump inhibitor—PPI)
SID	Strong ion difference
UTI	Urinary tract infection
